# Highly replicating hepatitis C virus variants emerge in immunosuppressed patients causing severe disease

**DOI:** 10.21203/rs.3.rs-6194507/v1

**Published:** 2025-06-09

**Authors:** Volker Lohmann, Paul Rothhaar, Christian Heuss, Margaret Tulessin, Zhiqing Wang, Ha Gyu-Thomas Seong, Colin Förster, Jocelyn Quistrebert, Haiting Chai, Marvin Reineke, Louise Benning, Jonathan Honegger, Maike Hofmann, Robert Thimme, Jörg Timm, Graham Cooke, Paul Schnitzler, Uta Merle, Naglaa H. Shoukry, Julie Bruneau, Chaturaka Rodrigo, Andrew Lloyd, Rowena Bull, Azim Ansari, Carolin Mogler, John McLauchlan, Xavier Forns, Sofía Pérez-del-Pulgar

**Affiliations:** Heidelberg University; Heidelberg University; Heidelberg University; Technical University of Munich; University of Oxford; Heidelberg University; Heidelberg University; University of Oxford; University of Oxford; University Hospital Heidelberg; University Hospital Heidelberg; The Ohio State University School of Medicine; University Hospital Freiburg; Department of Medicine II, University Hospital Freiburg, Freiburg, Germany.; Faculty of Medicine, University of Freiburg, Freiburg, Germany; Heinrich-Heine-University, University Hospital Düsseldorf; Imperial College London; Dept. of Virology, University of Heidelberg; Department of Internal Medicine IV, University Hospital Heidelberg, Heidelberg, Germany; Centre de Recherche du Centre hospitalier de l’Université de Montréal (CRCHUM); Centre de Recherche du CHUM; University of New South Wales; Kirby Institute, University of New South Wales; UNSW Sydney; University of Oxford; Institute of Pathology, TUM; University of Glasgow; University of Barcelona; University of Barcelona

## Abstract

Hepatitis C virus (HCV) exists as a heterogenous quasispecies, but the phenotypic consequences of viral variability are widely unexplored. Here we identified a replication enhancing domain (ReED) in nonstructural protein 5A conferring high replication fitness to clinical isolates. Accumulation of mutations in the ReED mediates high genome replication capacity. In a cohort of liver transplant patients, high replicator variants were exclusively found in individuals with severe disease outcome, suggesting that high viral replication fitness is associated with increased viral pathogenesis. Analysis of large sequence cohorts revealed that overall only 10% of viral genomes showed genetic signatures of high replicators, which were enriched in recipients of liver transplantations, patients developing hepatocellular carcinoma and in HIV coinfected individuals. Overall, our data suggests that low replication fitness is a hallmark of HCV, contributing to establishment of persistence, whereas high replicators appear to have an advantage under conditions of immune suppression, thereby enforcing pathogenesis.

## Introduction

Infections with the hepatitis C virus (HCV) become chronic in ~ 75% of all patients^1^, but direct acting antivirals (DAAs) achieve virus elimination in > 95% of cases, rendering chronic hepatitis C a curable disease (reviewed in^2^). Nevertheless, a protective vaccine remains elusive thus HCV still is a major public health concern with 50 million patients being infected and 240,000 dying from the disease in 2022^3^. Chronic HCV infections can last for decades but eventually end-stage liver disease or development of hepatocellular carcinoma (HCC) can create a need for liver transplantation (LTX). A severe complication post LTX in the context of HCV is the development of fibrosing cholestatic hepatitis (FCH) which is marked by high viral titers and rapid fibrosis progression accompanied by only mild inflammation^4^, occurring in 2–10% of LTX patients^5,6^. DAA treatment efficiency is also high in FCH patients^7,8^. HCV is a remarkably heterogenous virus with 8 genotypes (gt) and over 100 subtypes differing by up to 30% on a nucleotide sequence level^9^. This is enabled by the error prone genome replication of the virus resulting in a heterogenous group of viruses inhabiting each patient, the so called quasispecies^10^. This population structure helps the virus to escape the host immune response, but beyond that, phenotypic characterisations of HCV quasispecies evolution are scarce, one study reported a selection for variants with improved cell entry competence after LTX^11^.

On a molecular level, HCV has a positive sense RNA genome with untranslated regions (UTRs) flanking a single open reading frame. The resulting viral polyprotein is cleaved by host and viral proteases into 3 structural and 7 non-structural (NS) proteins. While the structural proteins core, E1 and E2 together with p7 and NS2 are crucial for infectious particle production, they are dispensable for genome replication, essentially involving NS3-NS5B and the UTRs (reviewed in^12^). Subgenomic replicons (SGRs) encompassing the HCV replicase, were established as the first HCV cell culture model based on the gt1b isolate Con1^13^. All isolates used for cell culture systems are based on consensus sequences derived from single patients, accounting for the risk of deleterious mutations being present in many individual viral clones due to the error prone replication process^14^. However, besides the gt2a isolate JFH1^15^, all wildtype (WT) isolates needed to acquire cell culture adaptive mutations to replicate in hepatoma cells commonly used in cell culture^16^. One hotspot for these adaptive mutations was found in NS5A^17^, a phosphoprotein without enzymatic function. Nevertheless, NS5A is involved in a variety of processes including determining the response to antiviral interferon treatment by accumulation of mutations in a 40 amino acid region termed interferon sensitivity determining region (ISDR)^18,19^. HCV cell culture systems were improved by the discovery that ectopic expression of the cytosolic lipid transporter SEC14L2 allowed for replication of WT isolates in cultured cells^20^. In this setup, we previously characterised a post-transplant gt1b isolate from an FCH patient, designated GLT1, which showed remarkably high replication fitness (RF) in cell culture^21^.

Here, we show that the high RF evolved in the GLT1 patient after LTX. This elevated RF was mediated by a region in NS5A including the ISDR where we identified accumulation of mutations as the sequence signature of high RF. Furthermore, we identified high RF as a general feature of HCV in FCH patients arguing for a direct connection between the increase in viral replication and the severe course of disease. Finally, we observed that sequence signatures of high RF are rare in immunocompetent patients but found more frequently in the context of profound immunosuppression, such as occurs at the early stages of transplantation, indicating a complex interplay between viral RF and the host immune response.

## Results

### HCV sequence evolution in a patient who received two liver transplantations

**Figure 1:**
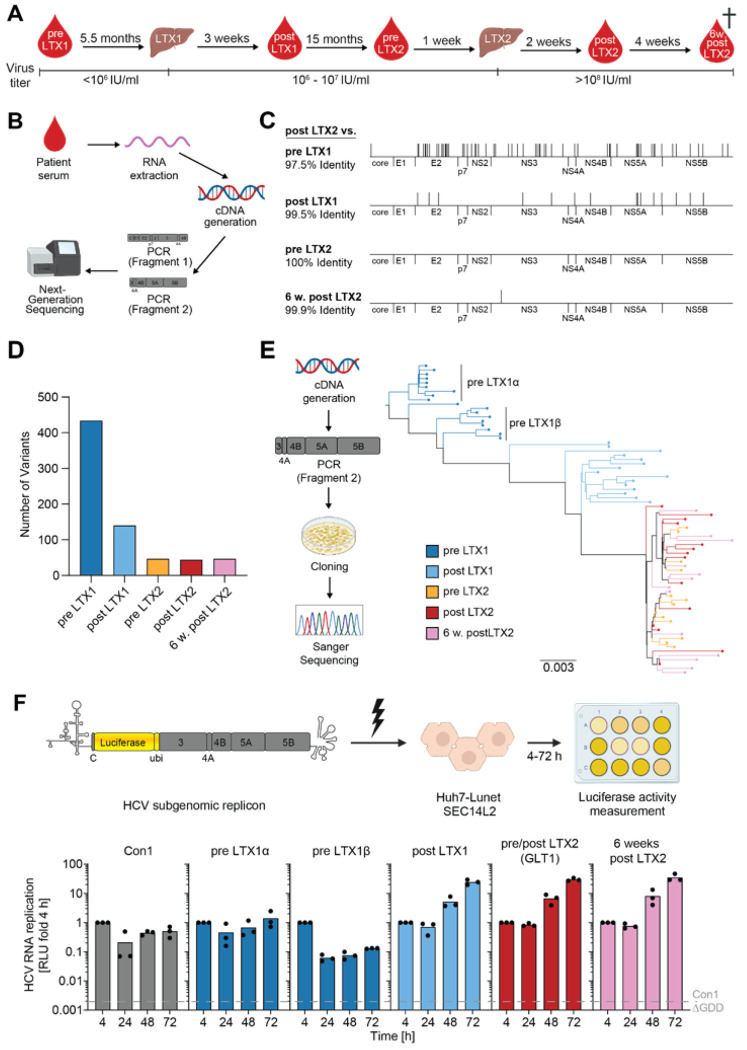
HCV evolution in the GLT1 patient. A) Schematic illustrating time points of collection and viral load of longitudinal serum samples from the GLT1 patient. Note that the patient passed away 6 weeks post LTX2. B) Schematic illustrating the workflow to retrieve bulk sequence information about HCV from patient sera. We extracted viral RNA from the serum, converted it into cDNA, amplified the HCV genome via PCR and performed next generation sequencing (NGS). For technical reasons, the HCV genome was amplified in two fragments spanning the entire coding sequence of the viral genome. C) Amino acid alignments of the consensus sequences at the indicated time points with the post LTX2 (=GLT1) sequence, differences are indicated by black dashes. D) Number of DNA variants (QD > 1) in the HCV population of each time point. E) Schematic of generation of sequencing data on the level of individual viral clones (left panel) and resulting phylogenetic tree for the second PCR fragment encompassing most of the viral replicase. F) SGRs of the indicated constructs were electroporated into Huh7-Lunet SEC14L2 cells (upper panel), luciferase activity in cell lysates (RLU) was quantified as a correlate of RNA replication efficiency at the given time points and normalised to 4 h to account for differences in transfection efficiency. Con1 ΔGDD served as a replication deficient negative control. Data are from three independent biological replicates measured in technical duplicates. Each dot depicts the result of one replicate and the bar indicates the mean of all replicates.

We recently characterised the GLT1 isolate which showed remarkably high RF in cell culture^21^. GLT1 was isolated from a patient who received two LTXs and represented the consensus sequence of the viral population after LTX2. To study the viral sequence evolution over the course of the two LTXs (Fig. 1A), we performed next generation sequencing (NGS) on two overlapping amplicons spanning the entire coding sequence of the genome (Fig. 1B). The most drastic change in the consensus sequence dominating at each time point was seen after LTX1 (Fig. 1C) where the HCV population became much more homogenous and remained so until the patient’s death (Fig. 1D). To gain a more comprehensive view on distinct viral subpopulations, we next analysed sequencing data from individually cloned viral genomes (Fig. 1E, left panel). In line with the NGS data, phylogenetic analysis revealed again a more heterogenous population structure pre LTX1 with at least two distinct HCV subspecies (Fig. 1E, S1A). Both the bulk sequencing data and the analysis of individual viral clones indicated LTX1 having a strong impact on the viral population while LTX2 did not have a substantial effect on the quasispecies.

To understand the phenotypic implications of the observed sequence evolution, we characterised changes in RF, cell entry and infectious particle production over the course of the two LTXs. Post LTX, we saw no selection for increased cell entry in experiments with HCV pseudoparticles (HCVpp) or improved infectious particle production by employing chimeric genomes (Fig. S1B&C). To assess RNA replication fitness, we constructed reporter SGRs^22^ harbouring the consensus sequence of the viral replicase (NS3-NS5B) for each timepoint (Fig. 1F, upper panel), and for both subspecies we found in the pre LTX1 sample (Fig. 1E). The resulting constructs were transfected into hepatoma cells expressing the lipid transporter SEC14L2 which is needed for replication of HCV WT isolates in cell culture^20^. Compared to the gt1b gold standard isolate Con1^13^, both subspecies pre LTX1 showed a similarly low RF (Fig. 1F, lower panel). Strikingly, all SGRs based on the post LTX time points showed more than 50-fold increase in RF arguing for a selection towards high RF in the context of LTX.

Increased RF after LTX argued for a prominent role of both the replication space provided by the new liver and the immunosuppression introduced with LTX1 in exerting evolutionary pressure on the HCV quasispecies. Thus, genome replication fitness is a so far overlooked major selection factor for HCV upon LTX.

### High replication fitness is mediated by a small region in NS5A

**Figure 2:**
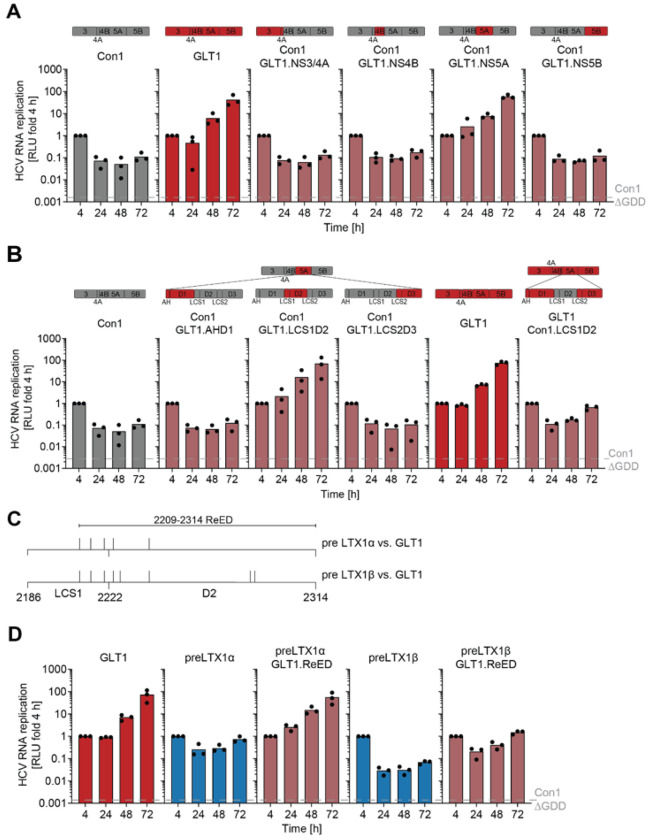
Viral determinants of high replication fitness. A, B, D) SGRs of the indicated constructs were electroporated into Huh7-Lunet SEC14L2 cells, luciferase activity in cell lysates (RLU) was quantified as a correlate of RNA replication efficiency at the given time points and normalised to 4 h to account for differences in transfection efficiency. Con1 ΔGDD served as a replication deficient negative control. Data are from three independent biological replicates measured in technical duplicates. Each dot depicts the result of one replicate and the bar indicates the mean of all replicates. C) Amino acid alignment of NS5A.LCS1D2 between GLT1 and the respective subspecies pre LTX1, differences are indicated by black dashes.

We next set out to identify mutations crucial for the increase in RF post LTX (Fig. 1F). To this end, we created chimeric Con1 SGRs (low replicator) where one protein of the replicase was replaced by its GLT1 (high replicator) counterpart (Fig. 2A, upper panel). Transfer of GLT1 NS5A into Con1 was sufficient to elevate the RF of Con1 by more than 100-fold (Fig. 2A, lower panel). Transfer of NS5A subdomains into Con1 (Fig. 2B, upper panel) identified Low Complexity Sequence 1 and Domain 2 (LCS1D2) of GLT1 NS5A as being sufficient to boost the RF of Con1 to the level of GLT1 (Fig. 2B, lower panel). LCS1D2 from Con1 further converted the high replicator GLT1 into a low replicator. These results argued for LCS1D2 being the main determinant of HCV RF. Splitting up LCS1D2 as well as further mapping of the region underlined that mutations all over LCS1D2 except the C-terminus contributed to its effects on RF (Fig. S2A&C). Therefore, we defined the Replication Enhancing Domain (ReED) to encompass the whole LCS1D2 region besides a highly conserved patch at the N-terminus (Fig. 2C). Interestingly, the ReED contains the ISDR, an interaction site with the essential host factor cyclophilin A^23^ and a disputed binding domain for protein kinase R (Fig. S2B)^24,25^.

In the GLT1 patient, we could see a high number of amino acid differences in the ReED between the two subspecies dominating pre LTX1 and GLT1 (Fig. 2C). To functionally characterize ReED evolution, we created chimeric SGRs of preLTX1α and preLTX1β containing the GLT1 ReED which dominated post LTX2. Indeed, the GLT1 ReED boosted replication of the two pre-LTX subspecies 76-fold and 22-fold, respectively (Fig. 2D), confirming that selection for mutations in the ReED resulted in the high RF post LTX in the GLT1 patient.

### Fibrosing cholestatic hepatitis is associated with highly replication fit HCV

**Figure 3:**
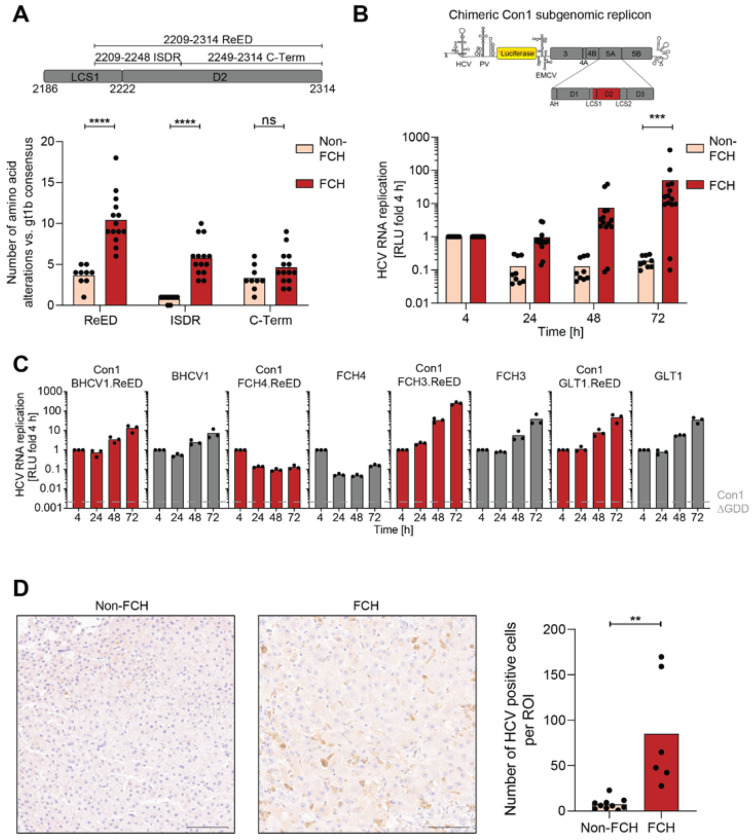
Genetic and phenotypic analysis of the FCH cohort. A) Amino acid deviations from the gt1b consensus sequence were counted for each patient sequence post LTX, an insertion was counted as one mutation, irrespective of its length. The analysed regions are depicted in a schematic (upper panel). Each dot depicts the result of one patient and the bar indicates the mean of all patients. B) Con1 based chimeric SGRs harbouring the ReED of a patient (upper panel) were electroporated into Huh7-Lunet SEC14L2 cells, luciferase activity in cell lysates (RLU) was quantified as a correlate of RNA replication efficiency 72 h after electroporation and normalised to 4 h to account for differences in transfection efficiency. Data are from three independent biological replicates measured in technical duplicates. Each dot represents the mean RLU of one patient and the bar indicates the mean of all non-FCH/ FCH patients. A&B) Statistical significance was determined with a Mann-Whitney-U-test. ns = not significant, *** = p < 0.001, **** = p < 0.0001. C) SGRs of the indicated constructs were electroporated into Huh7-Lunet SEC14L2 cells, luciferase activity in cell lysates (RLU) was quantified as a correlate of RNA replication efficiency at the given time points and normalised to 4 h to account for differences in transfection efficiency. Con1 ΔGDD served as a replication deficient negative control. Data are from three independent biological replicates measured in technical duplicates. Each dot depicts the result of one replicate and the bar indicates the mean of all replicates. D) Sections of human livers from post-transplant patients either developing FCH or not were stained via IHC with an HCV NS5A targeting antibody. Representative image from one non-FCH (left panel, patient non-FCH12) and one FCH patient (middle panel, patient FCH14), scale bar represents 100 μm. Quantification of HCV positive cells per 500 μm x 500 μm region (right panel). Six regions per patient were analysed. Each dot depicts the mean result of one patient and the bar indicates the mean of all non-FCH/FCH patients. Statistical significance was determined with a two-sided Student’s t-test. ** = p < 0.01.

To assess whether increased RF was a general feature of viral isolates evolving after LTX, we focused on a cohort of 22 additional HCV gt1b infected LTX patients of which 13 developed FCH post LTX^26,27^. Sequence analysis revealed a significantly higher divergence from the gt1b consensus for the ReED of FCH patients than for non-FCH patients, which was only driven by accumulation of mutations in the ISDR but not in the C-terminal part of the ReED (Fig. 3A). All FCH patients had 3 or more ISDR mutations while the non-FCH patients had a maximum of 1 ISDR mutation rendering accumulation of ISDR mutations a clear sequence signature of FCH (Fig. 3A; S3A). To assess the RF of the FCH and non-FCH patients in cell culture, we created chimeric Con1 SGRs encoding the ReEDs of the post LTX patients (Fig. 3B, upper panel). A chimera harbouring the gt1b consensus ReED, based on 358 gt1b full-length sequences, showed low RF, comparable to Con1 (Fig. S3B), indicating that the majority of hepatitis C patients is infected with a low replicator HCV. Strikingly, constructs harbouring the ReED from FCH patients replicated on average more than 100-fold higher than the non-FCH patient derived constructs, with only two exceptions (Fig. 3B; S3B). We verified that chimeric Con1 SGRs with patient-derived ReEDs accurately represented the RF of a patient’s HCV isolate, by comparing the RF of four chimeras with SGRs encoding the patient’s complete replicase (Fig. 3C) and confirmed the ReED’s effect on RF in full-length HCV genomes (Fig. S4A). Overall, our data argued for a connection between accumulation of mutations in the ISDR with high RF in cell culture and the highly pathogenic course of liver disease after LTX.

For FCH patients with available complete replicase sequences^21,26,27^, phylogenetic analysis showed no distinct FCH cluster indicating that most isolates could potentially convert into a high replicator and cause FCH by acquiring ReED mutations (Fig. S4B). The genetic diversity upon LTX decreased in almost all cases, irrespective of disease outcome (Fig. S5A). However, observations similar to GLT1 were noted in only 3 patients, where a low replicator variant evolved into a high replicator variant after LTX. In contrast, 7 FCH patients already exhibited a high replicator sequence before undergoing LTX. (Fig. S5C). Interestingly, torque teno virus (TTV) load in post LTX sera as a surrogate marker for immunocompetence^28,29^, revealed no differences between non-FCH and FCH patients (Fig. S5B). This highlights the complexity of HCV evolution but overall underlines that acquisition of a high replicator HCV variant in late chronic infection or upon LTX was decisive for FCH development.

All patients of the FCH cohort showed very high serum titers (> 10,000,000 IU/ml, Fig. S5D), a hallmark of FCH^4^, which we wanted to confirm on the level of HCV antigen load in the liver. We therefore used immunohistochemistry to compare the number of HCV positive cells in the liver of FCH and non-FCH patients. Indeed, HCV positive cells were significantly more abundant in liver samples of FCH patients, arguing for high intrahepatic viral replication (Fig. 3D, S6) which is in line with previous studies^30,31^. Interestingly, FCH4, containing three ISDR mutations post LTX (Fig. S3A), but having a low replicator phenotype in cell culture (Fig. 3C), showed exceptionally high antigen loads (Fig. S6B), indicative for a high replicator variant in vivo. This suggested that our assessment of RF in cell culture widely reflected replication competence in vivo but might have missed some high replicator variants. This data furthermore indicated that liver pathogenesis was directly or indirectly caused by high intrahepatic virus replication.

Overall, we could show a strong correlation between accumulation of ISDR mutations leading to increased viral RF and a severe course of disease for LTX patients.

### The ReED is a regulator of replication fitness in all major genotypes

**Figure 4:**
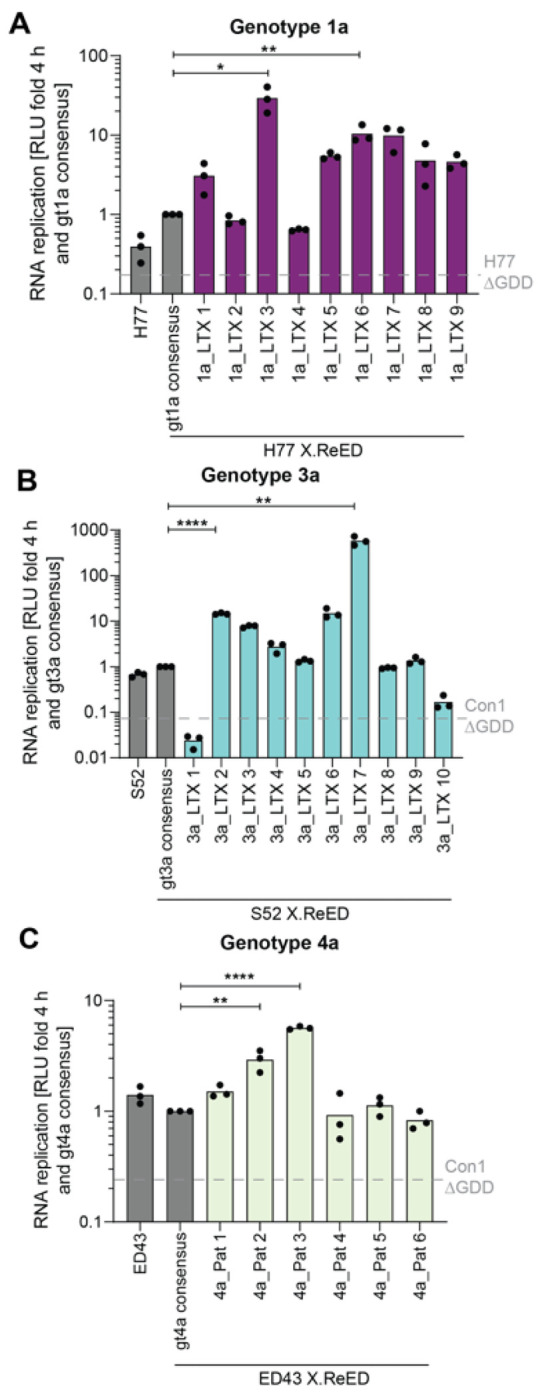
The ReED regulates RF in all major genotypes. All ReEDs tested were derived from LTX (gt1a, gt3a) or LTX and HCC (gt4a) patients from the HCV Research UK cohort^32^. SGRs of the indicated constructs were electroporated into Huh7-Lunet SEC14L2 cells, luciferase activity in cell lysates (RLU) was quantified as a correlate of RNA replication efficiency at the given time points and normalised to 4 h to account for differences in transfection efficiency and subsequently normalised to the values for the gt specific consensus of the respective replicate. H77 ΔGDD (A) or Con1 ΔGDD (B&C) served as a replication deficient negative control. Data are from three independent biological replicates measured in technical duplicates. Each dot depicts the result of one replicate and the bar indicates the mean of all replicates. Statistical significance for the two constructs with the highest RF per gt was determined with a two-sided Student’s t-test. ns = not significant, * = p < 0.05, ** = p < 0.01, **** = p < 0.0001.

Our study so far was focused on HCV gt1b. However, HCV is a very heterogenous virus with 8 gts and over 100 subtypes^9^. To address the impact of the ReED on RF of other major genotypes, we retrieved ReED sequences mainly from LTX patients infected with gt1a, gt3a or gt4a from the HCV Research UK cohort^32^. All sequences had at least 3 ISDR mutations when compared to the gt specific consensus sequence. To study the impact of these ReEDs on RF, we used established isolates derived from chimpanzee infectious clones as backbones for SGRs: H77 (gt1a)^33,34^, S52 (gt3a) and ED43 (gt4a)^35^. Patient derived ReED sequences as well as the gt specific consensus sequence were introduced into the respective isolates. RF of all constructs with a consensus ReED was low but was increased for ReEDs with altered ISDR in many cases (Fig. 4). The increases in RF were moderate compared to gt1b due to the cell culture systems being less suitable for non-gt1b isolates^36^ (reviewed in^14^). Still, for gt1a, 7 out of 9 isolates with altered ISDR replicated at least 2-fold higher than the consensus (Fig. 4A, Fig. S7A), while in gt3a 5 out of 10 (Fig. 4B, Fig. S7B) and in gt4a 2 out of 6 isolates showed this phenotype (Fig. 4C, Fig. S7C). Overall, our experiments revealed that the ReED can regulate HCV replication fitness of all major HCV genotypes.

### Frequency of potential high replicators in different clinical contexts

**Figure 5:**
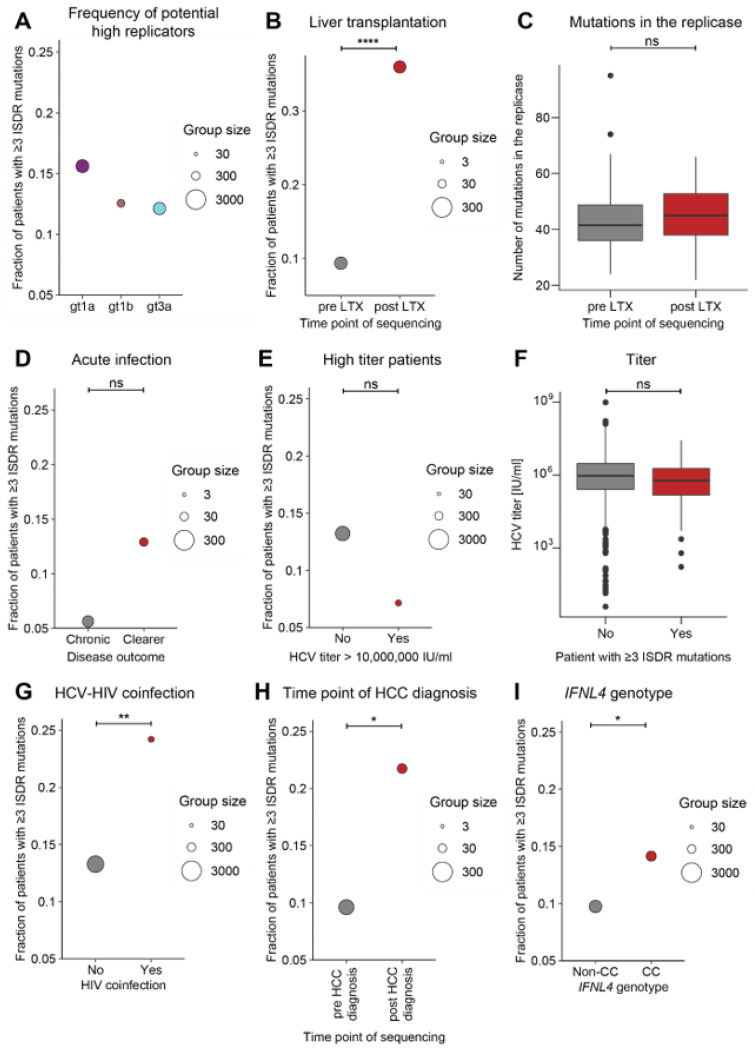
Sequence signatures of high RF in various clinical contexts. For each patient’s ISDR sequence, the number of amino acid differences compared to the gt specific consensus sequence was determined. If an ISDR had 3 or more mutations, the patient was considered to be infected with a potential high replicator. A-C, E-H) Data based on the HCV Research UK cohort^32^. A) Fraction of potential high replicators stratified by gt. B-I) Data for gts 1a, 1b and 3a was pooled, for all analyses the results for the individual gt had the same trend as the combined analysis. B) Fraction of potential high replicators in patients who ever received an LTX depending on whether samples for HCV sequencing were collected pre or post LTX. C) Number of mutations in the replicase (excluding the ReED) compared to the gt specific consensus in the patients depicted in B). D-I) Post LTX patients were excluded from the analysis to prevent confounding the results. D) Sequences from the HITS-p cohort^68^ combined with 28 patients participating in the Montreal Hepatitis C cohort (HEPCO)^88^ sequenced for this study, informing about the fraction of potential high replicators in acute patients who either developed a chronic HCV infection or cleared the virus without antiviral treatment. E) Relative abundance of potential high replicators in the context of very high serum titers (>10,000,000 IU/ml). F) Correlation of potential high replicators and HCV serum titers. 193 potential high replicators were compared to 1295 potential low replicators. The most recent titer measurement was used and only patients with an available titer measurement within a year of sequencing were included. G) Fraction of high replicators in patients with an HCV-HIV coinfection. H) Fraction of high replicators in patients who ever had an HCC diagnosis depending on whether samples for HCV sequencing were collected pre or post HCC diagnosis. I) Combined data from patients of the HCV Research UK^32^, STOPHCV^40^ and BOSON^39^ cohorts where host genomic sequence information was available, showing the fraction of potential high replicators depending on the host’s *IFNL4* rs12979860 genotype. Statistical significance was determined using a two-sided Student’s t-test (C&F) or a Fisher’s exact test (B, D, E, G-I). ns = not significant, * = p < 0.05, ** = p < 0.01, *** = p < 0.001, **** = p < 0.0001.

So far, by mainly focusing on LTX patients we could show that the ReED can regulate RF in all major gts with accumulation of ISDR mutations being the sequence signature of high RF. This sequence signature allowed us to investigate patients of the HCV Research UK cohort containing HCV sequence information and detailed clinical annotations for more than 2000 patients, mainly infected with the major HCV gts 1a, 1b and 3a^32^. For these gts, 12–15% of patients harboured a potential high replicator defined by having 3 or more amino acid mutations in the ISDR when compared to the respective gt specific consensus sequence (Fig. 5A). Because of the similar prevalence of potential high replicators, we decided to perform all following analyses with a combined dataset of gt 1a, 1b and 3a patients. In line with our previous results, we found a significant enrichment of potential high replicators in patients sequenced after LTX (Fig. 5B). The enrichment of mutations was also specific to the ISDR, with no significant accumulation of mutations in the whole replicase (Fig. 5C). Early acute infections should create similar conditions as LTX, offering a naïve liver in absence of adaptive immune pressure. This might create a selective advantage for high replicating variants but also trigger adaptive immunity more efficiently and thereby impact on infection outcome. Indeed, a higher proportion of potential high replicators was observed in patients who cleared the virus compared to patients who developed a chronic infection (Fig. 5D). Since samples from this disease stage are scarce due to the inapparent course of disease, no statistical significance was reached. Still, this data indicated that high RF might only be advantageous for HCV variants under conditions of immunosuppression, such as LTX, but rather facilitate clearance in immunocompetent individuals.

In FCH patients, HCV serum titers of more than 10,000,000 IU/ml were common, suggesting a correlation with RF in immunosuppressed patients (Fig. S5D). Surprisingly, in non-transplant patients from the HCV Research UK cohort, there was no trend towards enrichment of potential high replicators in patients with titers exceeding 10,000,000 IU/ml (Fig. 5E) and no significant overall correlation between titer and ISDR mutations was found (Fig. 5F). This lack of correlation was confirmed in a cohort of pregnant women^37^ (Fig. S8A), suggesting that HCV serum titers are not primarily determined by RF in immunocompetent patients.

In FCH patients, we saw a strong correlation between high RF and pathogenesis. But in non-LTX patients of the HCV Research UK cohort, no association between the presence of potential high replicators and the liver status at the time point of sequencing, liver stiffness or past diagnosis of cirrhosis or decompensation were observed (Fig. S8B-D), as for other parameters like gender, drug consumption, bilirubin levels, biological age or time since first infection (Fig. S8D; S9A&B). In contrast, HCV-HIV coinfection was a parameter significantly associated with increased presence of potential high replicators (Fig. 5G), which might again be linked to immunosuppressive conditions. Finally, in the subgroup of patients who ever had an HCC diagnosis in their life (Fig. S8D), we found that potential high replicators were significantly more common in patients after they received their HCC diagnosis (Fig. 5H). Thus, previous HCC diagnosis or HCV-HIV coinfection appear to be scenarios favouring the emergence of potential HCV high replicators.

To understand why only some patients harboured a high replicator, we turned towards host genetic determinants. In the context of the immune response to HCV, it was discovered that single nucleotide polymorphisms (SNPs) in the *IFNL4* gene coding for interferon lambda 4 (IFNλ4) are associated with disease outcome^38^. To this end, we analysed a cohort of 1481 patients with available host genetic and virus sequence information^32,39,40^. Here, we found that potential high replicators were more prevalent in patients with the CC genotype for IFNL4 SNP rs12979860, favouring spontaneous clearance (Fig. 5I). Beyond *IFNL4*, a genome wide association study did not reveal any other host genetic determinants significantly associated with the presence of potential high replicators (Fig. S9C). Thus, the importance of SNPs in the *IFNL4* gene further highlight the tight interplay of the immune response and RF.

Overall, our data highlight that the emergence of potential high replicators can correlate also with clinical parameters beyond the LTX context like HIV coinfection or HCC as well as the genetic background of the *IFNL4* gene.

## Discussion

In the present study, we identified a region within NS5A, termed ReED, conferring high genome replication fitness to clinical isolates of the main HCV genotypes. Accumulation of mutations in a portion of the ReED, known as ISDR, was the sequence signature of high replicator variants. HCV variants with high replicator signatures were enriched under conditions of immune suppression and associated with severe pathogenesis after LTX.

HCV pathogenesis is usually considered to be immune mediated^41^. It was previously speculated that direct cytopathic effects by HCV could be causative of FCH^42,43^, a hypothesis that is strongly supported by our data, showing 100-fold higher genome replication fitness of isolates dominating in FCH patients. To date, only increased cell entry competence was identified after LTX^11^, but our study demonstrates that high genome replication fitness can be another so far unrecognised selection factor, decisive for disease outcome. Interestingly, the presence of mutations enhancing viral fitness were also described for hepatitis B virus (HBV) patients developing FCH^44,45^. Since HCV associated FCH currently mainly occurs in the context of HCV negative patients receiving an HCV positive organ^46^, sequencing the viral quasispecies could allow an early identification of patients at risk.

On a sequence level, we were able to show that accumulation of mutations in the ISDR, which is the N-terminal part of the ReED, is a sequence signature of high RF. The ISDR was initially identified in the context of response to interferon (IFN) treatment in HCV gt1b infected patients in Japan^18^. The IFN sensitive “mutant type” was defined by at least 2 to 4 ISDR mutations^47–50^ which is in line with our cutoff value of 3 or more ISDR mutations being associated with FCH and high RF. Previous studies already suggested increased replication for gt1b by ISDR mutations^51,52^, but were limited by a low magnitude of effects in the implemented cell culture models. Our study now highlights the general importance of the ReED in dramatically increasing RF of the major gts 1a, 1b, 3a and 4a, drawing on samples from a diverse range of geographical backgrounds.

While all dominant HCV variants in FCH patients had high replicator sequence signatures, FCH4&9 showed no high replicator phenotype in cell culture. Since FCH4 had the highest HCV antigen load in the liver and both isolates were associated with very high serum titers, it appears likely that they still represent high replicators in vivo. FCH4&9 had a relatively high genetic diversity compared to the whole FCH cohort. Thus, divergent high replicator subtypes in the quasispecies might have remained unrecognised in the available consensus sequence. It furthermore remains elusive how the ReED affects RF, but domain 2 of NS5A which encompasses the C-terminal 80% of the ReED, was shown to interact with the HCV RNA dependent RNA polymerase NS5B, modulating polymerase activity^53–55^. The variability of independent substitution and insertion patterns observed in high replicator variants overall renders a loss of function more likely than a gain of function. We therefore hypothesize that the consensus ReED is a negative regulator of NS5B activity with the mutations observed in high replicators unleashing a constitutively active NS5B. However, further studies are required to understand how the ReED governs HCV replication fitness on a molecular level.

New replication space provided by the transplanted liver combined with suppression of adaptive immunity are plausible determinants providing a selective advantage for high replicator variants. HCV associated FCH was also diagnosed in kidney^46^, heart^56^, lung^57^ and hematopoietic stem cell^58^ transplants and AIDS patients^59^, arguing for immunosuppression as the main driver of evolution towards high RF. HCV-HIV coinfection is an additional condition with increased proportion of potential high replicator variants identified in our study, that might be linked to immunosuppression due to AIDS (reviewed in^61^). Additionally, potential high replicators were more frequent in patients after receiving their HCC diagnosis which could be linked to immunosuppressive effects of cancer treatment or the tumor microenvironment (reviewed in^62^) although a cohort with more detailed clinical background on the patients would be needed to support this hypothesis. Our observation is in line with previous reports on higher frequency of altered ISDRs in HCC patients^50,60^ and FCH development upon chemotherapy^61^.

While weakened adaptive immunity might favour high replicator variants, it cannot fully explain the presence of high replicators in immunocompetent patients. Here, ISDR mutations increasing RF might compensate for the fitness costs of resistant variants upon DAA therapy or of immune escape variants emerging in chronic infection. The enrichment of HCV variants with high replicator signature in patients with the defective *IFNL4* CC allele we found in our cohort is further pointing to the complex interplay of viral replication fitness and host immunity. The CC allele was shown to favour cure upon IFN treatment and clearance of acute infections^38,62^, since IFNλ4 hinders antigen presentation and the adaptive immune response^63^. We further found potential high replicators more frequently in acute patients who went on to clear the infection. HCV with high RF might be more vulnerable to the adaptive immunity due to higher antigen expression and therefore more likely being cleared in fully immunocompetent hosts. Along these lines, HCV clearance after waning of immune checkpoint inhibitors for cancer treatment^64^ or after reduction of immunosuppressive therapy in two FCH patients^65,66^ were reported elsewhere. Viral replication fitness thereby appears to be a key determinant of infection outcome. This needs to be considered for the choice of the infection inoculum used in controlled human infection models, currently planned to facilitate development of protective HCV vaccines^67^.

In conclusion, our study shows that HCV RF is a selection factor particularly in immunocompromised patients. We identified a key regulator of RF within the HCV genome and could show a massively elevated RF in FCH patients, decisive for disease outcome. Our data thereby establish genome replication fitness as a new important variable in the interplay between HCV and the immune system potentially contributing to clearance or persistence of the infection, with further potential implications for DAA treatment and vaccine development.

## Materials & Methods

### Ethics statement

This study was approved by the ethics committee of the medical faculty of Heidelberg University (ethics votes: S-399/2012, S-720/2022 and S-743/2023). 12 liver samples were provided by the tissue bank of the German Center for Infection Research (DZIF, Heidelberg, Germany) in accordance with the regulations of the tissue bank and the approval of the ethics committee of Heidelberg University (ethics vote: S-399/2012). One liver sample of an HCV negative patient suffering from cholestatic liver disease was approved by the ethics committee of Klinikum rechts der Isar (MRI, Munich) (ethics vote: 518/19 S). The Montreal Hepatitis C Cohort study (HEPCO) is approved by the Research Ethics Committee of the Centre de Recherche du Centre Hospitalier de l’Université de Montréal (CRCHUM) (Approval number: SL 05.014). The studies were conducted in accordance with the local legislation and institutional requirements. The participants provided their written informed consent to participate in this study.

### Patients

The HCV Research UK cohort^32^, the cohort of pregnant women^37^, the HITS-p cohort^68^, the HEPCO^69^, the GLT1 patient^21^, the BHCV1 patient^27^, patients FCH1-12 and non-FCH1-3^26^ were described previously. Liver samples of patients non-FCH10-19 and FCH13&14 were provided by the tissue bank of the German Center for Infection Research (DZIF, Heidelberg, Germany) and liver samples from patients FCH1, 4, 5 and 12 are from a previously described cohort^26^. Patients non-FCH4-9 were selected to be post LTX without FCH diagnosis and no successful antiviral treatment in the first year post LTX. Patients FCH1-12 and non-FCH1-9 are described in more detail in Table S2.

### Cell culture

Clones of the immortalised hepatoma cell line Huh7-Lunet either ectopically expressing CD81, SEC14L2 or an empty plasmid conferring blasticidin resistance were described previously^21^. They were cultured in Dulbecco’s modified Eagle’s medium (Gibco), supplemented with 1% (v/v) non-essential amino acids, 2 mM L-glutamine, 100 U/ml penicillin, 100 mg/ml streptomycin, 10% (v/v) heat-inactivated fetal-calf serum (FCS) and 5 μg/ml blasticidin. All cell lines were regularly tested to check they were free of mycoplasma contamination using the MycoAlert Mycoplasma Detection kit (Lonza).

### Cloning

If not specified otherwise, DNA fragments for cloning were either generated via PCR using the PhusionFlash High-Fidelity Master Mix (Thermo Fisher Scientific), via digest with appropriate restriction enzymes (New England Biolabs, Thermo Fisher Scientific) or via DNA synthesis (BioCat, Thermo Fisher Scientific). DNA fragments were combined either via T4 DNA ligase (Thermo Fisher Scientific) or with the NEBuilder HiFi DNA assembly cloning kit (New England Biolabs). Correct DNA sequence of the final plasmid was confirmed via Sanger or Oxford Nanopore sequencing (Microsynth AG). All plasmids used and generated during this study can be found in Table S3.

### Sequencing of viral RNA extracted from patient sera

Viral RNA was purified from 200 μl patient serum using the NucleoSpin Virus kit (Macherey-Nagel). cDNA based on the extracted RNA was generated using the SuperScript IV First-Strand Synthesis kit (Invitrogen) with HCV genotype specific primers (Table S1). To amplify the DNA, nested PCR was performed using the SuperFi II DNA polymerase kit (Invitrogen) according to the manufacturer’s instructions. A first 50 μl PCR reaction (primers see Table S1) with 25% of the cDNA as template was run for 30 cycles. 5 μl of this PCR product was used as template for the second PCR (primers see Table S1) running again for 30 cycles. The desired amplicon was purified via gel electrophoresis with the NucleoSpin Gel and PCR Clean-up kit (Macherey-Nagel). Fragments were sequenced either via Sanger sequencing (Microsynth AG) or NGS was performed using the MiSeq v2 (2x250bp) platform (Illumina) with an average sequencing depth of ~ 4000 reads (Microsynth AG).

### Analysis of NGS data

Sequence reads were aligned to the GLT1 WT reference sequence (GenBank accession OM222702) using the Burrows-Wheeler aligner^70^. Variant calling was performed using the HaplotypeCaller of the genome analysis toolkit^71^. Consensus sequences were derived with the consensus function of bcftools^72^.

### Phylogenetic analysis of individual viral clones

To retrieve individual viral clones from the GLT1 patient, the same amplicons used to derive the consensus sequences were cloned into a pCR-XL-2-TOPO vector using the TOPO^™^ XL-2 Complete PCR Cloning Kit (Invitrogen). Phylogenetic trees were constructed using the minimum evolution principle^73^ with the tools of the virus and pathogen database and analysis resource (VipR)^74^. Trees were visualised using FigTree.

### In vitro transcription (IVT)

DNA plasmids were linearised with appropriate restriction enzymes cutting after the 3’UTR of the respective construct. DNA was purified using the NucleoSpin Gel and PCR Clean-up kit (Macherey-Nagel). IVT was performed by combining 10 μg linearised plasmid DNA, 6 μl T7 polymerase (homemade), 12.5 μl of rNTP solution (25 mM of each rNTP), 20 μl 5x RRL buffer (1 M HEPES, pH 7.5, 1 M MgCl2, 1 M Spermidine and 1 M DTT), 100 U rRNasin RNase inhibitor (Promega) and filling up to 100 μl with water. The reaction was incubated at 37°C overnight. 20 U RQ1 DNAse (Promega) was added the next day and incubated for 1 h at 37°C. RNA was purified via phenol-chloroform extraction. Here, the reaction mixture was combined with 420 μl H_2_O, 60 μl 2 M sodium acetate (pH = 4.5) and 400 μl phenol/water saturated (pH < 5). After mixing and incubation on ice for 10 min, samples were centrifuged at 14,800 rpm, 4°C for 10 min. Supernatant was transferred into a new tube and mixed with one volume chloroform. The mixture was centrifuged for 10 min at 14,800 rpm. Supernatant was again transferred into a new tube and mixed with 0.7 volumes isopropanol and subsequently centrifuged for 10 min at 14,800 rpm. Supernatant was removed, the RNA washed with 70% ethanol and resuspended in water. RNA quality was controlled via agarose gel electrophoresis.

### Electroporation

To transfect hepatoma cells with IVT RNA, electroporation was performed. After detaching the cells from the cell culture dish, they were washed once with PBS. Then, cells were resuspended in Cytomix (120 mM KCl, 0.15 mM CaCl_2_, 10 mM K_2_HPO_4_/KH_2_PO_4_ (pH = 7.6), 2 mM EGTA, 25 mM HEPES, 5 mM MgCl_2_ and freshly added 2 mM ATP, 5 mM glutathione) to reach a final concentration of 10^7^ cells/ml. Of this suspension, 200 μl (2*10^6^ cells) were combined with 2.5 μg of the respective RNA to be transfected in an electroporation cuvette (gap width: 0.2 cm) and electroporated at 975 μF and 166 V using a GenePulser system (BioRad). Electroporated cells were then resuspended in 6 ml DMEM. For the timepoints to be harvested 4 h and 24 h after electroporation, 1 ml of this mixture was put in a well of a twelve-well plate. For the 48 h and 72 h time points, 0.5 ml of the mixture and 0.5 ml of medium were combined in a well. Cells were then incubated at 37°C and 5% CO_2_.

### Luciferase assay

Samples were harvested by washing with PBS and subsequent lysis in 200 μl lysis buffer (1% Triton X-100, 25 mM glycyl glycine, 15 mM MgSO_4_, 4 mM EGTA, and freshly added 1 mM DTT) per well. Firefly luciferase activity measurements were performed by mixing 80 μl lysate per technical replicate with 350 μl assay buffer (25 mM glycyl glycine, 15 mM K_3_PO_4_ buffer (pH = 7.8), 0.15 M MgSO_4_, 4 mM EGTA (pH = 7.8) and freshly added 1 mM DTT, 2 mM ATP). During luminescence measurement, a Lubat LB9510 tube luminometer (Berthold Technologies) injected 100 μl of a luciferin solution (0.2 mM luciferin, 25 mM glycyl glycine) into the sample and then detected the signal for 20 s. Nano luciferase measurements were performed with the same device using the Nano-Glo luciferase assay system (Promega) according to the manufacturer’s instructions.

### Particle production

To produce infectious particles, Huh7-Lunet CD81 cells were electroporated with IVT RNA as described above. One ml of the resulting suspension of electroporated cells was combined with 1 ml fresh medium and seeded in a 6-well plate. After 72 h, supernatant was filtered (0.45 μm) and added to naïve Huh7-Lunet CD81 cells, seeded the day prior at a density of 1.5*10^5^ cells in a 6-well plate. Cells were washed at 4 h post infection and at 72 h post infection, infected cells were harvested for luciferase assay as described above.

### HCVpp

HIV-based particles bearing HCV envelope proteins (HCVpp) were used for studies of cell entry. For transfection, a mix of 2.16 μg pcDNA based envelope protein expression construct^75^, 6.42 μg HIV gag-pol expression construct pCMVΔ8.74^76^, 6.42 μg firefly luciferase transducing retroviral vector^77^, 45 μl polyethyleneimine (PEI) and 740 μl Opti-MEM was created, vortexed for 10 s and incubated at RT for 20 min before being added to 1.2*10^6^ HEK293T cells which were seeded the day prior in a 6 cm-diameter-dish. Medium was replaced after 6 h. 48 h after transfection, supernatant containing the pseudoparticles was passed through a 0.45 μm filter and used to infect 4*10^4^ naïve Huh7-Lunet CD81 cells seeded in a 12-well plate the day before. After 72 h, harvesting of samples and luciferase assay were performed as described above. To quantify HCVpp titers used for infection, SYBR Green based Product Enhanced Reverse Transcriptase assay (SG-PERT) was performed^78^ using the Takyon SYBR green kit (Eurogentec).

### Sequence analysis

Sequence alignments were performed using ClustalOmega through the msa package^79^ and visualised via Jalview^80^. Based on 358 (gt1b)^21^, 496 (gt1a), 509 (gt3a), 29 (gt4a) HCV amino acid sequences encompassing the whole viral polyprotein retrieved from ViPR, genotype specific consensus sequences were determined using EMBOSS cons^81^. The 358 gt1b sequences were also used to generate the phylogenetic tree of gt1b using the minimum evolution principle.

To analyse sequence cohorts, each patient’s ISDR amino acid sequence was aligned to the genotype specific consensus sequence and the number of differences was determined automatically through a script in R.

### Immunohistochemistry

Slides (thickness 2–3 μm) from 16 HCV infected human cases were examined. They were stained with haematoxylin and eosin (H&E) according to the standard protocol at the Institute of Pathology, Technical University Munich (TUM). Slides were then independently evaluated by two experienced liver and comparative pathologists. Immunohistochemistry (IHC) was performed using the Bond RXm autostainer system (Leica) with all reagents supplied by Leica. Tissue sections were first deparaffinised using the Leica de-wax kit to remove embedding medium, followed by rehydration through a graded alcohol series with decreasing ethanol concentrations (100%, 96%, 70%). Heat-induced epitope retrieval was performed using epitope retrieval solution 1 (H1), corresponding to a citrate buffer at pH = 6, for 30 minutes. Endogenous peroxidase activity was blocked with 3% H_2_O_2_. The tissue sections were incubated with the primary antibody (Hepatitis C virus immunostaining - HCV 9E10; 1:4000) at room temperature for 15 min. Antibody detection was carried out using the Polymer Refine Detection Kit. Visualization of antibody binding was achieved using the chromogenic substrate diaminobenzidine (DAB; Medac Diagnostica). Analysis was performed using Aperio ImageScope (Leica Biosystems). For each patient, in 6 regions of interest (500 x 500 μm) HCV positive cells were counted, regions of interest were selected to contain at least one HCV positive cell.

### Analysis of genetic determinants

Genetic determinants were analysed based on a previously described cohort^82^. Blood specimens collected at enrolment were used to generate host-genotyping data through the Affymetrix UK Biobank array, which covers ~ 820,000 markers. Quality control exclusion of SNPs using PLINK v1.9^83^ consisted of: minor allele frequency (MAF) < 5%, call rate of < 98%, Hardy-Weinberg equilibrium p < 10^− 5^, batch difference and sex difference in allele frequency (SNPs with a significant different MAF, p < 10^− 5^, were filtered out). A total of 542,730 high-quality autosomal SNPs were retained for imputation. Individuals with a call rate < 95% and had an outlier heterozygosity value (± 3 SD from the same self-identified ancestry group’s heterozygosity rate mean) were excluded. Sex of each remaining subject was verified by comparing the reported sex with the observed sex based on X chromosome method-of-moments F coefficient. Pairwise genotype concordance for all patients was assessed to identify duplicates. Patients with genotype concordance > 95% at a set of pruned SNPs were considered duplicates and the members of the pairs with the lower call rate were excluded. Identity-by-descent (IBD) analysis was done to detect duplicated samples. Imputation was performed on the TOPMed Imputation Server using the TOPMed reference panel version r2^84^. ~6.3 million of SNPs with an imputation quality score greater than 0.3 were retained for genetic analyses.

We conducted a principal component analysis (PCA) to evaluate population substructure and map self-identified racial/ethnic groups onto the estimated principal components (PCs). Kinship coefficients were estimated to select unrelated individuals using PC-Relate, as implemented in the R package GENESIS^85^. The SNPRelate package in R^86^ was then used for PCA on pruned common genotyped SNPs and using a set of unrelated individuals, defined as pairwise kinship coefficients less than 2^− 9/2^. These 2 estimation procedures were iterated to ensure that the kinship coefficients were unbiased in admixed individuals and that the PCs were computed over unrelated individuals. For the viral data, PCA was performed on the nucleotide data as follows. Tri- and quad-allelic sites were converted to binary variables, and the amino acid frequencies were standardised to have mean zero and unit variance. MATLAB (release 2015a, The MathWorks) was used to perform the PCA using the singular value decomposition function.

To test for association between human SNPs and HCV mutation count in ISDR region, we performed logistic regression using PLINK2^87^, adjusted for the human population structure (ten first PCs) and the virus population structure (five first PCs). Age, gender, and virus sequencing center were also included as covariates to control the potential confounding.

### Determination of TTV titers

TTV quantification was carried out using the TTV R-Gene assay (BioMérieux), a real-time PCR assay that targets the TTV 5’UTR. This assay has a dynamic range from 250 to 10^9^ copies/ml, with a detection limit of 250 copies/ml. TTV DNA was extracted from serum samples using the QIAsymphony SP platform (QIAGEN), and PCR was performed on a Light Cycler 480 Instrument II (Roche Diagnostics). The viral load was determined using a standard curve.

### Statistical analysis

Statistical analyses were performed using GraphPad Prism (Graphpad Software) or R. For numerical data, a two-tailed Student’s t-test was applied when normal distribution was assumed; otherwise, the Mann-Whitney U test was utilised. For categorial values, Fisher’s exact test was used.

## Data Availability

Parts of the FCH cohort were previously published under the accession numbers MK092096- MK092105 (FCH1-5), MK092106-MK092111 (Non-FCH1-3), OM222702 (GLT1) and HQ719473 (BHCV1). Sequence information on all other FCH patients generated during this study is deposited under the following accessions: PV083181-PV083206. NGS data from the GLT1 patient are deposited in the sequence read archive (SRA) under the accession PRJNA1214216.

## References

[1] Hajarizadeh B., Grebely J. & Dore G.J. Epidemiology and natural history of HCV infection. Nat. Rev. Gastroenterol. Hepatol (2013). 10.1038/nrgastro.2013.107 23817321

[2] Manns M.P., Hepatitis C virus infection. Nature Reviews Disease Primers 3, 17006 (2017). 10.1038/nrdp.2017.6 28252637

[3] WHO. Global hepatitis report 2024: action for access in low- and middle-income countries, (World Health Organization, Geneva, 2024).

[4] Wiesner R.H., Sorrell M. & Villamil F. Report of the First International Liver Transplantation Society Expert Panel Consensus Conference on Liver Transplantation and Hepatitis C. Liver Transplantation 9, S1–S9 (2003). 10.1053/jlts.2003.50268 14586888

[5] Narang T.K., Ahrens W. & Russo M.W. Post–liver transplant cholestatic hepatitis C: A systematic review of clinical and pathological findings and application of consensus criteria. Liver Transplantation 16, 1228–1235 (2010). 21031537 10.1002/lt.22175

[6] Hori T., Fibrosing cholestatic hepatitis C in post-transplant adult recipients of liver transplantation. Ann Gastroenterol 29, 454–459 (2016). 27708510 10.20524/aog.2016.0069 PMC5049551

[7] Leroy V., Efficacy of Sofosbuvir and Daclatasvir in Patients With Fibrosing Cholestatic Hepatitis C After Liver Transplantation. Clinical Gastroenterology and Hepatology 13, 1993–2001.e1992 (2015). 26044317 10.1016/j.cgh.2015.05.030

[8] Forns X., Sofosbuvir compassionate use program for patients with severe recurrent hepatitis C after liver transplantation. Hepatology 61(2015). 10.1002/hep.27681 25557906

[9] Vo-Quang E. & Pawlotsky J.-M. ‘Unusual’ HCV genotype subtypes: origin, distribution, sensitivity to direct-acting antiviral drugs and behaviour on antiviral treatment and retreatment. Gut 73, 1570–1582 (2024). 38782565 10.1136/gutjnl-2024-332177 PMC11347264

[10] Domingo E. & Perales C. Viral quasispecies. PLOS Genetics 15, e1008271 (2019). 31622336 10.1371/journal.pgen.1008271 PMC6797082

[11] Fafi-Kremer S., Viral entry and escape from antibody-mediated neutralization influence hepatitis C virus reinfection in liver transplantation. Journal of Experimental Medicine 207, 2019–2031 (2010). 20713596 10.1084/jem.20090766 PMC2931157

[12] Lohmann V. Hepatitis C virus RNA replication. Curr. Top. Microbiol. Immunol 369, 167–198 (2013). 23463201 10.1007/978-3-642-27340-7_7 PMC7122439

[13] Lohmann V., Replication of subgenomic hepatitis C virus RNAs in a hepatoma cell line. Science 285, 110–113 (1999). 10390360 10.1126/science.285.5424.110

[14] Lohmann V. Hepatitis C virus cell culture models: an encomium on basic research paving the road to therapy development. Medical microbiology and immunology 208, 3–24 (2019). 30298360 10.1007/s00430-018-0566-x

[15] Kato T., Efficient replication of the genotype 2a hepatitis C virus subgenomic replicon. Gastroenterology 125, 1808–1817 (2003). 14724833 10.1053/j.gastro.2003.09.023

[16] Lohmann V., Hoffmann S., Herian U., Penin F. & Bartenschlager R. Viral and cellular determinants of hepatitis C virus RNA replication in cell culture. J. Virol 77, 3007–3019 (2003). 12584326 10.1128/JVI.77.5.3007-3019.2003 PMC149776

[17] Blight K.J., Kolykhalov A.A. & Rice C.M. Efficient initiation of HCV RNA replication in cell culture. Science 290, 1972–1974 (2000). 11110665 10.1126/science.290.5498.1972

[18] Enomoto N., Comparison of full-length sequences of interferon-sensitive and resistant hepatitis C virus 1b. Sensitivity to interferon is conferred by amino acid substitutions in the NS5A region. The Journal of Clinical Investigation 96, 224–230 (1995). 7542279 10.1172/JCI118025 PMC185192

[19] Enomoto N., Mutations in the Nonstructural Protein 5a Gene and Response to Interferon in Patients with Chronic Hepatitis C Virus 1b Infection. New England Journal of Medicine 334, 77–82 (1996). 8531962 10.1056/NEJM199601113340203

[20] Saeed M., SEC14L2 enables pan-genotype HCV replication in cell culture. Nature 524, 471–475 (2015). 26266980 10.1038/nature14899 PMC4632207

[21] Heuss C., A Hepatitis C virus genotype 1b post-transplant isolate with high replication efficiency in cell culture and its adaptation to infectious virus production in vitro and in vivo. PLOS Pathogens 18, e1010472 (2022). 35763545 10.1371/journal.ppat.1010472 PMC9273080

[22] Frese M., Interferon-gamma inhibits replication of subgenomic and genomic hepatitis C virus RNAs. Hepatology 35, 694–703 (2002). 11870386 10.1053/jhep.2002.31770

[23] Dujardin M., Cyclophilin A allows the allosteric regulation of a structural motif in the disordered domain 2 of NS5A and thereby fine-tunes HCV RNA replication. Journal of Biological Chemistry 294, 13171–13185 (2019). 31315928 10.1074/jbc.RA119.009537 PMC6721930

[24] Gale M. Jr., Control of PKR protein kinase by hepatitis C virus nonstructural 5A protein: molecular mechanisms of kinase regulation. Mol Cell Biol 18, 5208–5218 (1998). 9710605 10.1128/mcb.18.9.5208 PMC109106

[25] Francois C., Expression of hepatitis C virus proteins interferes with the antiviral action of interferon independently of PKR-mediated control of protein synthesis. Journal Of Virology 74, 5587–5596 (2000). 10823866 10.1128/jvi.74.12.5587-5596.2000 PMC112046

[26] Gambato M., Hepatitis C virus intrinsic molecular determinants may contribute to the development of cholestatic hepatitis after liver transplantation. Journal of General Virology 100, 63–68 (2019). 30451649 10.1099/jgv.0.001175

[27] Koutsoudakis G., Cell Culture Replication of a Genotype 1b Hepatitis C Virus Isolate Cloned from a Patient Who Underwent Liver Transplantation. PloS one 6, e23587 (2011). 21887279 10.1371/journal.pone.0023587 PMC3160967

[28] Görzer I., Pre-Transplant Plasma Torque Teno Virus Load and Increase Dynamics after Lung Transplantation. PloS one 10, e0122975 (2015). 25894323 10.1371/journal.pone.0122975 PMC4404260

[29] Ruiz P., Torque Teno Virus Is Associated With the State of Immune Suppression Early After Liver Transplantation. Liver Transpl 25, 302–310 (2019). 30375165 10.1002/lt.25374

[30] Fenwick F., Immunohistochemical assessment of hepatitis C virus antigen in cholestatic hepatitis after liver transplantation. Journal of Clinical Pathology 59, 174–178 (2006). 16443734 10.1136/jcp.2005.028126 PMC1860319

[31] Mensa L., Imaging of hepatitis C virus infection in liver grafts after liver transplantation. Journal of Hepatology 59, 271–278 (2013). 23548198 10.1016/j.jhep.2013.03.023

[32] McLauchlan J., Cohort Profile: The Hepatitis C Virus (HCV) Research UK Clinical Database and Biobank. Int J Epidemiol 46, 1391–1391h (2017). 28338838 10.1093/ije/dyw362 PMC5837619

[33] Kolykhalov A.A., Transmission of hepatitis C by intrahepatic inoculation with transcribed RNA. Science 277, 570–574 (1997). 9228008 10.1126/science.277.5325.570

[34] Yanagi M., Purcell R.H., Emerson S.U. & Bukh J. Transcripts from a single full-length cDNA clone of hepatitis C virus are infectious when directly transfected into the liver of a chimpanzee. Proceedings of the National Academy of Sciences 94, 8738–8743 (1997). 10.1073/pnas.94.16.8738 PMC23104 9238047

[35] Gottwein J.M., Novel infectious cDNA clones of hepatitis C virus genotype 3a (strain S52) and 4a (strain ED43): genetic analyses and in vivo pathogenesis studies. J. Virol 84, 5277–5293 (2010). 20200247 10.1128/JVI.02667-09 PMC2863810

[36] Costa R., SEC14L2, a lipid-binding protein, regulates HCV replication in culture with inter- and intra-genotype variations. J Hepatol 70, 603–614 (2019). 30472319 10.1016/j.jhep.2018.11.012

[37] Coss S.L., CD4+ T cell restoration and control of hepatitis C virus replication after childbirth. The Journal of Clinical Investigation 130, 748–753 (2020). 31904583 10.1172/JCI123623 PMC6994162

[38] Thomas D.L., Genetic variation in IL28B and spontaneous clearance of hepatitis C virus. Nature 461, 798–801 (2009). 19759533 10.1038/nature08463 PMC3172006

[39] Foster G.R., Efficacy of sofosbuvir plus ribavirin with or without peginterferon-alfa in patients with hepatitis C virus genotype 3 infection and treatment-experienced patients with cirrhosis and hepatitis C virus genotype 2 infection. Gastroenterology 149, 1462–1470 (2015). 26248087 10.1053/j.gastro.2015.07.043

[40] Cooke G.S., Strategic treatment optimization for HCV (STOPHCV1): a randomised controlled trial of ultrashort duration therapy for chronic hepatitis C. Wellcome Open Res 6, 93 (2021). 34405118 10.12688/wellcomeopenres.16594.1 PMC8361811

[41] Morozov V.A. & Lagaye S. Hepatitis C virus: Morphogenesis, infection and therapy. World J Hepatol 10, 186–212 (2018). 29527256 10.4254/wjh.v10.i2.186 PMC5838439

[42] Ramírez S., Pérez-Del-Pulgar S. & Forns X. Virology and pathogenesis of hepatitis C virus recurrence. Liver transplantation : official publication of the American Association for the Study of Liver Diseases and the International Liver Transplantation Society 14 Suppl 2, S27–35 (2008). 18825723 10.1002/lt.21644

[43] McCaughan G.W. & Zekry A. Impact of immunosuppression on immunopathogenesis of liver damage in hepatitis C virus-infected recipients following liver transplantation. Liver Transplantation 9, S21–S27 (2003). 14586891 10.1053/jlts.2003.50269

[44] Angus P.W., Hepatitis B virus precore mutant infection is associated with severe recurrent disease after liver transplantation. Hepatology 21, 14–18 (1995). 7806147

[45] Kumar R. Review on hepatitis B virus precore/core promoter mutations and their correlation with genotypes and liver disease severity. World J Hepatol 14, 708–718 (2022). 35646275 10.4254/wjh.v14.i4.708 PMC9099108

[46] Kapila N., Al-Khalloufi K., Bejarano P.A., Vanatta J.M. & Zervos X.B. Fibrosing cholestatic hepatitis after kidney transplantation from HCV-viremic donors to HCV-negative recipients: A unique complication in the DAA era. American Journal of Transplantation 20, 600–605 (2020). 31448549 10.1111/ajt.15583

[47] Pascu M., Sustained virological response in hepatitis C virus type 1b infected patients is predicted by the number of mutations within the NS5A-ISDR: a meta-analysis focused on geographical differences. Gut 53, 1345–1351 (2004). 15306598 10.1136/gut.2003.031336 PMC1774214

[48] Shirakawa H., Pretreatment prediction of virological response to peginterferon plus ribavirin therapy in chronic hepatitis C patients using viral and host factors. Hepatology 48, 1753–1760 (2008). 18925643 10.1002/hep.22543

[49] Fukuhara T., Mutations in hepatitis C virus genotype 1b and the sensitivity of interferon-ribavirin therapy after liver transplantation. Journal of Hepatology 52, 672–680 (2010). 20346532 10.1016/j.jhep.2009.10.034

[50] El-Shamy A., Polymorphisms of the core, NS3, and NS5A proteins of hepatitis C virus genotype 1b associate With development of hepatocellular carcinoma. Hepatology 58, 555–563 (2013). 23281009 10.1002/hep.26205

[51] Kohashi T., Site-specific mutation of the interferon sensitivity-determining region (ISDR) modulates hepatitis C virus replication. Journal of Viral Hepatitis 13, 582–590 (2006). 16907844 10.1111/j.1365-2893.2006.00739.x

[52] Ikeda M., Yi M., Li K. & Lemon S.M. Selectable subgenomic and genome-length dicistronic RNAs derived from an infectious molecular clone of the HCV-N strain of hepatitis C virus replicate efficiently in cultured Huh7 cells. J. Virol 76, 2997–3006 (2002). 11861865 10.1128/JVI.76.6.2997-3006.2002 PMC135991

[53] Ngure M., Interactions of the Disordered Domain II of Hepatitis C Virus NS5A with Cyclophilin A, NS5B, and Viral RNA Show Extensive Overlap. ACS Infect Dis 2, 839–851 (2016). 27676132 10.1021/acsinfecdis.6b00143

[54] Shirota Y., Hepatitis C virus (HCV) NS5A binds RNA-dependent RNA polymerase (RdRP) NS5B and modulates RNA-dependent RNA polymerase activity. J. Biol. Chem 277, 11149–11155 (2002). 11801599 10.1074/jbc.M111392200

[55] Bessa L.M., NMR reveals the intrinsically disordered domain 2 of NS5A protein as an allosteric regulator of the hepatitis C virus RNA polymerase NS5B. J Biol Chem 292, 18024–18043 (2017). 28912275 10.1074/jbc.M117.813766 PMC5672029

[56] Plana A., Belkin M. & Grinstein J. (455) Acute Liver Injury as a Complication of Heart Transplant from a Hepatitis C+ Donor. The Journal of Heart and Lung Transplantation 42, S210 (2023).

[57] Aleyadeh W., Outcomes of early vs late treatment initiation in solid organ transplantation from hepatitis C virus nucleic acid test-positive donors to hepatitis C virus-uninfected recipients: Results from the HCV-TARGET study. American Journal of Transplantation 24, 468–478 (2024). 37871798 10.1016/j.ajt.2023.10.006

[58] Evans A.T., Fibrosing Cholestatic Hepatitis C After Hematopoietic Cell Transplantation: Report of 3 Fatal Cases. The American Journal of Surgical Pathology 39, 212–220 (2015). 25517948 10.1097/PAS.0000000000000345

[59] Rosenberg P.M., Rapidly progressive fibrosing cholestatic hepatitis-hepatitis C virus in HIV coinfection. The American Journal of Gastroenterology 97, 478–483 (2002). 11866292 10.1111/j.1572-0241.2002.05459.x

[60] Giménez-Barcons M., High amino acid variability within the NS5A of hepatitis C virus (HCV) is associated with hepatocellular carcinoma in patients with HCV-1b–related cirrhosis. Hepatology 34, 158–167 (2001). 11431747 10.1053/jhep.2001.25512

[61] Pellicelli A.M., Cholestatic hepatitis C after chemotherapy containing rituximab in diffuse large B cell lymphoma. Annals of Hepatology 14, 756–761 (2015). 26256907

[62] Ge D., Genetic variation in IL28B predicts hepatitis C treatment-induced viral clearance. Nature 461, 399–401 (2009). 19684573 10.1038/nature08309

[63] Chen Q., Interferon lambda 4 impairs hepatitis C viral antigen presentation and attenuates T cell responses. Nature Communications 12, 4882 (2021). 10.1038/s41467-021-25218-x PMC8360984 34385466

[64] Wilson H., Macdonald D. & Bryce K. Clearance of Hepatitis C Virus following Immune Checkpoint Inhibitor Therapy for Hepatocellular Carcinoma: Case Report. Case Rep Gastroenterol 18, 347–351 (2024). 39015527 10.1159/000539646 PMC11250384

[65] Gow P.J. & Mutimer D. Successful outcome of liver transplantation in a patient with hepatitis C and common variable immune deficiency. Transplant International 15, 380–383 (2002). 12122517 10.1007/s00147-002-0420-2

[66] Li D.L., Fang J., Zheng Z., Wu W. & Wu Z. Successful Treatment of Fibrosing Cholestatic Hepatitis Following Kidney Transplantation With Allogeneic Hematopoietic Stem Cell Transplantation: A Case Report. Medicine 94, e480 (2015). 25654389 10.1097/MD.0000000000000480 PMC4602713

[67] Liang T.J., Challenge Inoculum for Hepatitis C Virus Controlled Human Infection Model. Clinical Infectious Diseases 77, S257–S261 (2023). 37579208 10.1093/cid/ciad336 PMC10681659

[68] Cunningham E.B., Ongoing incident hepatitis C virus infection among people with a history of injecting drug use in an Australian prison setting, 2005-2014: The HITS-p study. J Viral Hepat 24, 733–741 (2017). 28256027 10.1111/jvh.12701

[69] Grebely J., Hajarizadeh B. & Dore G.J. Direct-acting antiviral agents for HCV infection affecting people who inject drugs. Nature Reviews Gastroenterology & Hepatology 14, 641–651 (2017). 28831184 10.1038/nrgastro.2017.106

[70] Li H. & Durbin R. Fast and accurate short read alignment with Burrows–Wheeler transform. Bioinformatics 25, 1754–1760 (2009). 19451168 10.1093/bioinformatics/btp324 PMC2705234

[71] Van der Auwera G.A., From FastQ Data to High-Confidence Variant Calls: The Genome Analysis Toolkit Best Practices Pipeline. Current Protocols in Bioinformatics 43, 11.10.11–11.10.33 (2013). 10.1002/0471250953.bi1110s43 PMC4243306 25431634

[72] Danecek P., Twelve years of SAMtools and BCFtools. Gigascience 10 (2021). 10.1093/gigascience/giab008 PMC7931819 33590861

[73] Desper R. & Gascuel O. Fast and accurate phylogeny reconstruction algorithms based on the minimum-evolution principle. J Comput Biol 9, 687–705 (2002). 12487758 10.1089/106652702761034136

[74] Pickett B.E., ViPR: an open bioinformatics database and analysis resource for virology research. Nucleic Acids Res 40, D593–598 (2012). 22006842 10.1093/nar/gkr859 PMC3245011

[75] Schaller T., Analysis of hepatitis C virus superinfection exclusion by using novel fluorochrome gene-tagged viral genomes. J. Virol 81, 4591–4603 (2007). 17301154 10.1128/JVI.02144-06 PMC1900174

[76] Dull T., A third-generation lentivirus vector with a conditional packaging system. J Virol 72, 8463–8471 (1998). 9765382 10.1128/jvi.72.11.8463-8471.1998 PMC110254

[77] Koutsoudakis G., Characterization of the early steps of hepatitis C virus infection by using luciferase reporter viruses. J. Virol 80, 5308–5320 (2006). 16699011 10.1128/JVI.02460-05 PMC1472176

[78] Vermeire J., Quantification of reverse transcriptase activity by real-time PCR as a fast and accurate method for titration of HIV, lenti- and retroviral vectors. PloS one 7, e50859 (2012). 23227216 10.1371/journal.pone.0050859 PMC3515444

[79] Bodenhofer U., Bonatesta E., Horejš-Kainrath C. & Hochreiter S. msa: an R package for multiple sequence alignment. Bioinformatics 31, 3997–3999 (2015). 26315911 10.1093/bioinformatics/btv494

[80] Waterhouse A.M., Procter J.B., Martin D.M.A., Clamp M. & Barton G.J. Jalview Version 2—a multiple sequence alignment editor and analysis workbench. Bioinformatics 25, 1189–1191 (2009). 19151095 10.1093/bioinformatics/btp033 PMC2672624

[81] Madeira F., The EMBL-EBI search and sequence analysis tools APIs in 2019. Nucleic Acids Res 47, W636–W641 (2019). 30976793 10.1093/nar/gkz268 PMC6602479

[82] Ansari M.A., Genome-to-genome analysis highlights the effect of the human innate and adaptive immune systems on the hepatitis C virus. Nature Genetics 49, 666–673 (2017). 28394351 10.1038/ng.3835 PMC5873514

[83] Purcell S., PLINK: A Tool Set for Whole-Genome Association and Population-Based Linkage Analyses. The American Journal of Human Genetics 81, 559–575 (2007). 17701901 10.1086/519795 PMC1950838

[84] Das S., Next-generation genotype imputation service and methods. Nature Genetics 48, 1284–1287 (2016). 27571263 10.1038/ng.3656 PMC5157836

[85] Gogarten S.M., Genetic association testing using the GENESIS R/Bioconductor package. Bioinformatics 35, 5346–5348 (2019). 31329242 10.1093/bioinformatics/btz567 PMC7904076

[86] Zheng X., A high-performance computing toolset for relatedness and principal component analysis of SNP data. Bioinformatics 28, 3326–3328 (2012). 23060615 10.1093/bioinformatics/bts606 PMC3519454

[87] Chang C.C., Second-generation PLINK: rising to the challenge of larger and richer datasets. GigaScience 4(2015). 10.1186/s13742-015-0047-8 PMC4342193 25722852

[88] Grebely J., Cohort profile: the international collaboration of incident HIV and hepatitis C in injecting cohorts (InC3) study. Int J Epidemiol 42, 1649–1659 (2013). 23203695 10.1093/ije/dys167 PMC3887561

[89] Pietschmann T., Construction and characterization of infectious intragenotypic and intergenotypic hepatitis C virus chimeras. Proc. Natl. Acad. Sci. U. S. A 103, 7408–7413 (2006). 16651538 10.1073/pnas.0504877103 PMC1455439

[90] Esser-Nobis K., Analysis of hepatitis C virus resistance to silibinin in vitro and in vivo points to a novel mechanism involving nonstructural protein 4B. Hepatology 57, 953–963 (2013). 23322644 10.1002/hep.26260 PMC3593759

[91] Schult P., microRNA-122 amplieffis hepatitis C virus translation by shaping the structure of the internal ribosomal entry site. Nature Communications 9, 2613 (2018). 10.1038/s41467-018-05053-3 PMC6031695 29973597

[92] Blight K.J., McKeating J.A., Marcotrigiano J. & Rice C.M. Efficient replication of hepatitis C virus genotype 1a RNAs in cell culture. J. Virol 77, 3181–3190 (2003). 12584342 10.1128/JVI.77.5.3181-3190.2003 PMC149761

